# Nociceptive Biology of Molluscs and Arthropods: Evolutionary Clues About Functions and Mechanisms Potentially Related to Pain

**DOI:** 10.3389/fphys.2018.01049

**Published:** 2018-08-03

**Authors:** Edgar T. Walters

**Affiliations:** Department of Integrative Biology and Pharmacology, McGovern Medical School, The University of Texas Health Science Center at Houston, Houston, TX, United States

**Keywords:** nociceptive sensitization, nociceptor, hyperalgesia, allodynia, nerve injury, synaptic potentiation, anxiety, aversive learning

## Abstract

Important insights into the selection pressures and core molecular modules contributing to the evolution of pain-related processes have come from studies of nociceptive systems in several molluscan and arthropod species. These phyla, and the chordates that include humans, last shared a common ancestor approximately 550 million years ago. Since then, animals in these phyla have continued to be subject to traumatic injury, often from predators, which has led to similar adaptive behaviors (e.g., withdrawal, escape, recuperative behavior) and physiological responses to injury in each group. Comparisons across these taxa provide clues about the contributions of convergent evolution and of conservation of ancient adaptive mechanisms to general nociceptive and pain-related functions. Primary nociceptors have been investigated extensively in a few molluscan and arthropod species, with studies of long-lasting nociceptive sensitization in the gastropod, *Aplysia*, and the insect, *Drosophila*, being especially fruitful. In *Aplysia*, nociceptive sensitization has been investigated as a model for aversive memory and for hyperalgesia. Neuromodulator-induced, activity-dependent, and axotomy-induced plasticity mechanisms have been defined in synapses, cell bodies, and axons of *Aplysia* primary nociceptors. Studies of nociceptive sensitization in *Drosophila* larvae have revealed numerous molecular contributors in primary nociceptors and interacting cells. Interestingly, molecular contributors examined thus far in *Aplysia* and *Drosophila* are largely different, but both sets overlap extensively with those in mammalian pain-related pathways. In contrast to results from *Aplysia* and *Drosophila*, nociceptive sensitization examined in moth larvae (*Manduca*) disclosed central hyperactivity but no obvious peripheral sensitization of nociceptive responses. Squid (*Doryteuthis*) show injury-induced sensitization manifested as behavioral hypersensitivity to tactile and especially visual stimuli, and as hypersensitivity and spontaneous activity in nociceptor terminals. Temporary blockade of nociceptor activity during injury subsequently increased mortality when injured squid were exposed to fish predators, providing the first demonstration in any animal of the adaptiveness of nociceptive sensitization. Immediate responses to noxious stimulation and nociceptive sensitization have also been examined behaviorally and physiologically in a snail (*Helix*), octopus (*Adopus*), crayfish (*Astacus*), hermit crab (*Pagurus*), and shore crab (*Hemigrapsus*). Molluscs and arthropods have systems that suppress nociceptive responses, but whether opioid systems play antinociceptive roles in these phyla is uncertain.

## Introduction

Darwin (1871) considered pain an emotion that evolved by natural selection and is shared by many species. While most research addressing pain has focused on humans and a few mammalian species, findings shedding light on pain-related functions have also been made in invertebrate taxa. Many of these findings came from studies of species in Mollusca and Arthropoda. By species number, these are by far the two largest metazoan phyla, and they contain species with the most complex nervous systems and most sophisticated behavior of any invertebrates.

If human pain is a product of evolution, its neural and molecular mechanisms are unlikely to have arisen *de novo* in our species, and thus at least some processes important for human pain should also occur in other taxa. Informative comparisons and contrasts of pain-related phenomena across taxa require a clear definition of pain. Having primarily been investigated within a clinical/preclinical tradition, the most frequently cited definition of pain is from the International Association for the Study of Pain^1^: pain is “an unpleasant sensory and emotional experience associated with actual or potential tissue damage.” This definition has three distinctive features: (1) pain sensation is usually produced by noxious events that produce or threaten to produce injury, (2) the sensation includes sensory information about the noxious event (quality, location, intensity, etc.), and (3) the sensation is tied to a negative emotion that motivates immediate and future avoidance of the apparent source of the sensation (Walters, 2018). Aspects of each of these features can appear in responses to noxious stimuli in non-human species, including molluscan and arthropod species. One property of pain-like states that cannot be assessed conclusively in non-human animals is their emotional content, at least when emotion is defined in terms of conscious experience, as it often is (Izard, 2009). That is because subjective feeling is not directly accessible to observers of non-verbal organisms (Allen, 2004). However, the objective motivational effects that pain-like states have on behavior can be determined experimentally. It is likely that the behavioral consequences of pain-like motivational states were the major selection pressures for the evolution of pain mechanisms.

Pain-like states are inferred in animals from animal behavior and from neural activity in nociceptive systems that process information related to actual or imminent bodily injury. Noxious stimuli are detected by sensory neurons called primary nociceptors, and their activation (nociception) evokes defensive responses (Walters, 1994; Tobin and Bargmann, 2004; Smith and Lewin, 2009; Sneddon, 2015; Burrell, 2017). Because of the potency of nociceptors in driving both human pain and pain-like responses in animals (discussed in Odem et al., 2018), including selected molluscs and arthropods, and because enhanced function of nociceptors contributes substantially to various persistent pain states in mammals (Gold and Gebhart, 2010; Walters, 2012), a major focus of this article is on primary nociceptors.

## Immediate Responses to Noxious Stimulation in Gastropod Molluscs

To protect their soft bodies, most molluscs produce a hard shell, but many lack a shell or enough of a shell for adequate protection and must rely on other defenses. Among the seven extant taxonomic classes of molluscs, only two have been studied extensively by behavioral scientists and neurobiologists: Gastropoda and Cephalopoda, both of which include many species possessing little or no shell. The gastropods represent 80% of molluscan species and occupy an enormous range of marine, freshwater, and terrestrial habitats. Within Mollusca, only the coleoid cephalopods (octopus, cuttlefish, and squid) have more complex nervous systems and behaviors. Selected cephalopods and gastropods first attracted the attention of neuroscientists because their giant axons and neuronal somata permitted cellular studies that, until a few decades ago, were impossible in mammals. From the 1960s through the 1990s numerous laboratories exploited the experimental advantages of uniquely identifiable neurons in central neural circuits of gastropods to directly relate cellular and synaptic properties to the organization and mediation of defensive, feeding, and reproductive behaviors (Kandel, 1979; Chase, 2002). Unusual advantages include large neuronal somata (cell bodies) that (1) exhibit overshooting action potentials, (2) allow high-fidelity somal monitoring of synaptic potentials, and (3) display exceptional tolerance for prolonged or repeated impalement by micropipettes.

### Behavioral Responses to Noxious Stimulation in Gastropod Molluscs

Many mechanistic studies have focused on synaptic alterations underlying aversive learning and memory in the large marine snail, *Aplysia californica* (Kandel, 2001), which possesses only a rudimentary, internal shell that provides little or no protection. Associated behavioral studies of learning in *Aplysia* utilized electric shock to the soft body surface to modify behavior. Such shock was considered aversive because it evoked the same immediate defensive responses as produced either by strong mechanical pinch delivered to the body by an experimenter (which produced signs of tissue injury), by bites during staged attacks from a predatory gastropod, *Pleurobranchaea californica*, or by application of a chemical stimulus, NaCl crystals, to the skin (Walters and Erickson, 1986; Walters, 1994; Gasull et al., 2005). Noxious stimuli produced local withdrawal, directed release of ink and other defensive secretions, and escape locomotion directed away from the point of “attack” (Walters and Erickson, 1986). These responses are examples of active defenses that are common throughout the animal kingdom (Edmunds, 1974; Kavaliers, 1988; Walters, 1994): most notably, withdrawal, retaliation (in this case by directed ejection of offending chemicals) (Kicklighter et al., 2005; Love-Chezem et al., 2013), and flight. Production of defensive responses in *Aplysia* is accompanied by inhibition of competing behavioral responses (Walters et al., 1981; Illich et al., 1994; Acheampong et al., 2012).

### Nociceptors That Detect Noxious Stimulation in Gastropod Molluscs

Although electric shock is an artificial stimulus, shock delivered to the body surface of *Aplysia* evokes strong defensive responses indistinguishable from those activated by natural stimuli because the shock activates peripheral axons of the same primary nociceptors that are activated by noxious mechanical pressures (Walters et al., 1983a; Illich and Walters, 1997). Important functional properties of identified nociceptors in *Aplysia* (Walters et al., 1983a, 2004; Frost et al., 1997; Illich and Walters, 1997) – especially a relatively high threshold for activation by mechanical stimuli, and silence in the absence of noxious stimulation – are typical of mechano-nociceptors described in diverse animals, including leech (Nicholls and Baylor, 1968), lamprey (Martin and Wickelgren, 1971), teleost fish (Ashley et al., 2007); frog (Hamamoto and Simone, 2003), snake (Liang and Terashima, 1993), chicken (Koltzenburg and Lewin, 1997), mouse (Koltzenburg et al., 1997), rat (Handwerker et al., 1987), cat (Burgess and Perl, 1967), and monkey (Perl, 1968).

The nociceptors identified in *Aplysia* have coiled peripheral terminals embedded in the muscle layer rather than the skin (Steffensen and Morris, 1996), which can explain why sharp poking or pinching stimuli produce optimal activation, and light, brushing stimuli are ineffective. Unlike the nociceptors in insects discussed below, these neurons have somata located within central ganglia, far from their more vulnerable peripheral terminals. These nociceptors show functional properties (Walters et al., 1983a, 2004; Illich and Walters, 1997) more similar to mechanosensitive nociceptors in mammals that are myelinated, rapidly conducting, and rapidly adapting (Aδ- and Aβ-nociceptors) than to unmyelinated, slowly conducting and slowly adapting, often polymodal (chemosensitive) C-nociceptors (Light et al., 1992; Djouhri and Lawson, 2004). Myelin does not occur in molluscs (Roots, 2008), so increased conduction velocity depends upon increased axonal diameter. *Aplysia* nociceptors have central cell bodies and axonal diameters that, while not large compared to axons of truly giant neurons in *Aplysia* (Rayport et al., 1983; Steffensen et al., 1995), are much larger than the small axons coming from the far more numerous afferent neurons of unknown function that possess peripheral cell bodies (Xin et al., 1995). Relatively rapid conduction in *Aplysia* nociceptors and rapid adaptation are functionally consistent with rapid detection of the onset of threatening peripheral stimulation rather than provision of continuing information to the CNS about ongoing (e.g., inflammatory) noxious states, which in mammals is primarily provided by C-nociceptors (Odem et al., 2018). It is not known whether any of the small-diameter afferents or other sensory neurons in *Aplysia* have functions equivalent to those of mammalian C-fiber nociceptors – especially, the non-accommodating activity continuously induced by persistent states of injury and/or inflammation. Among all invertebrates, the leech N lateral neurons are the only nociceptors shown to have non-accommodating, polymodal properties (as well as weak capsaicin sensitivity) resembling the properties of mammalian C-fiber nociceptors (Pastor et al., 1996).

## Nociceptive Sensitization and Pain-Like States in Gastropod Molluscs

In mammals, an unusual property of nociceptive systems is a propensity to sensitize rather than adapt to repeated stimulation (Light et al., 1992; Walters, 1994). Nociceptive sensitization in mammals can also be produced by a single noxious event, which is often manifested as a pain-like response to a subsequent stimulus that normally would not be painful (allodynia) or as an enhanced response to a normally painful stimulus (hyperalgesia). As illustrated by the examples from gastropod molluscs discussed below, nociceptive sensitization can be central or peripheral and short-term or long-term, it includes both general sensitization and site-specific sensitization, and it can refer either to sensitized behavior or to sensitized neurons (for definitions, see Walters, 1994). Long-term sensitization probably protects animals made more vulnerable by serious injury during prolonged recuperative periods (Walters, 1994, 2012).

### General Sensitization in Gastropod Molluscs

Nociceptive sensitization has been studied more extensively in *Aplysia* than any other invertebrate. Most mechanistic studies have used a general sensitization paradigm, where noxious shock applied to one part of the body produces sensitization of withdrawal reflexes evoked by test stimuli applied to another body part (the sensitization occurs generally across the body). A single shock produces short-term general sensitization lasting hours, whereas multiple shocks spaced over hours or days produce long-term general sensitization lasting days or weeks (Carew et al., 1971; Pinsker et al., 1973). General sensitization can be induced by extrinsic neuromodulators (notably serotonin, 5-HT) released during noxious stimulation (Brunelli et al., 1976; Glanzman et al., 1989; Marinesco et al., 2004b). General sensitization in *Aplysia* was modeled at the cellular level by culturing nociceptors with motor neurons known to produce withdrawal responses *in vivo* and stimulating the culture with repeated application of the neuromodulator, 5-HT, to induce long-lasting (24–48 h or longer) facilitation of the synapses between these neurons. This simple model enabled the discovery of basic memory mechanisms that helped Eric Kandel win a Nobel Prize in 2000. The mechanisms by which 5-HT induces long-term presynaptic facilitation include highly conserved cell signaling pathways that also are involved in persistent pain in mammals (Walters and Moroz, 2009; Rahn et al., 2013; Byrne and Hawkins, 2015). Prominent among these are requirements during the induction or maintenance of long-term facilitation for signaling by cAMP and PKA (Brunelli et al., 1976; Castellucci et al., 1980; Ocorr et al., 1986; Bergold et al., 1992), PKCs (Sossin et al., 1994; Sutton et al., 2004; Cai et al., 2011), MAPK (Martin et al., 1997; Sharma et al., 2003), and tyrosine kinases (Purcell et al., 2003; Pu et al., 2014), as well as activation of the transcription factors CREB (Dash et al., 1990; Kaang et al., 1993; Liu et al., 2011) and C/EBP (Alberini et al., 1994; Herdegen et al., 2014), and regulation of local protein synthesis at the synapse by yet another protein kinase, target of rapamycin (TOR) (Casadio et al., 1999; Weatherill et al., 2010) and by cytoplasmic polyadenylation element binding protein (CPEB) (Miniaci et al., 2008).

The cellular model of general, long-term sensitization in *Aplysia* (5-HT applied to cultured nociceptors and motor neurons) has revealed important roles for non-coding RNAs in the regulation of gene expression in nociceptors. These include 5-HT-induced downregulation of micro RNAs (miR-124 and miR-22) (Rajasethupathy et al., 2009; Fiumara et al., 2015) and upregulation of Piwi-associated RNAs (piRNAs) (Rajasethupathy et al., 2012), which alter gene transcription, mRNA translation, and enzyme expression (e.g., increased presynaptic expression of atypical PKC) to enhance synaptic transmission from nociceptors.

Non-coding RNAs were suggested recently to mediate the transfer of sensitization from *Aplysia* receiving repeated noxious electric shock to unshocked recipients by injection of RNA extracted from central ganglia of shocked donors into the recipients (Bédécarrats et al., 2018). This surprising study is notable for several reasons. First, it suggests that extracellular RNAs (presumably non-coding RNAs) can promote behavioral and neuronal sensitization and thus might be yet another of the myriad extracellular signals that produce nociceptive sensitization (Walters, 2014; Ji et al., 2016). Second, the presumed non-coding RNAs are specific to the noxious event; RNAs extracted from the ganglia of unshocked *Aplysia* did not produce sensitization. Third, the donor RNA extract modestly increased the excitability of dissociated nociceptors, suggesting that extracellularly transported non-coding RNAs can directly sensitize nociceptors (perhaps by epigenetic alteration of gene expression by appropriate DNA methylation, as suggested by the authors’ finding that the RNA transfer effects were blocked by a DNA methylation inhibitor). The authors assume that the transferred non-coding RNA is part of the memory (“engram”) of sensitization and they imply that this RNA comes from the population of nociceptors they studied plus their downstream neural circuits, thus storing a central neural memory of the shock. However, the sensitization-specific RNA might be produced within any neurons (or other cell types) strongly activated (directly or indirectly) by the noxious shock, including numerous unidentified afferents with peripheral cell bodies that send their axons (which might transport RNA) to central ganglia (Xin et al., 1995). Injected RNAs would have access to central and peripheral neurons. Furthermore, the RNA-induced hyperexcitability of dissociated nociceptors they describe is unlikely to explain the observed enhancement of defensive siphon withdrawal because the siphon responses were elicited by weak tactile stimuli that are unlikely to activate this family of nociceptors (see above and Illich and Walters, 1997; Walters et al., 2004). Thus, their test stimuli, like the weak tactile test stimuli used in many *Aplysia* sensitization studies (e.g., Pinsker et al., 1973; Hawkins et al., 1998; Sutton et al., 2001; Cai et al., 2011), reveal an *allodynia*-like effect that is more likely to involve enhanced responsiveness of low-threshold mechanoreceptors than sensitization of the nociceptors that have been examined electrophysiologically. On the other hand, the interesting nociceptor hyperexcitability observed by Bédécarrats et al. (2018) suggests that use of an additional test stimulus that activates the nociceptors might reveal RNA transfer of a *hyperalgesia*-like effect (see also Walters, 1987a,b).

### Site-Specific Sensitization in Gastropod Molluscs

Most pain research in mammals is more concerned with the localized sensitization that occurs near a site of noxious stimulation or injury than with general sensitization expressed at distant sites (Woolf and Walters, 1991). Sensitization of *Aplysia* tail and siphon withdrawal responses elicited at a site that had received a brief series of electric shocks was found to be much stronger and longer lasting than general sensitization produced by the same shocks (Walters et al., 1983b; Walters, 1987b). This site-specific sensitization also occurred at a site of tissue injury, and thus appears functionally similar to primary hyperalgesia in mammals (Walters, 1987b). Both general sensitization and site-specific sensitization were linked to concurrent enhancement of synaptic connections from primary nociceptors to motor neurons mediating defensive reflexes, and to hyperexcitability of the nociceptor soma (Brunelli et al., 1976; Walters et al., 1983b; Frost et al., 1985; Scholz and Byrne, 1987; Cleary et al., 1998). These electrophysiological alterations were especially pronounced in tests of site-specific sensitization (Walters, 1987a). Importantly, short-term and long-term behavioral sensitization were found after staged attacks on *Aplysia* by lobsters, showing that both forms of sensitization can be induced by trauma resulting from interaction with a natural predator (Watkins et al., 2010; Mason et al., 2014). Lobster attacks produced long-term hyperexcitability (LTH) of nociceptor somata, but synaptic facilitation was not observed (Watkins et al., 2010; Mason et al., 2014).

Site-specific sensitization is produced by activity-dependent enhancement of the modulatory effects of extrinsic neuromodulators including 5-HT (Carew et al., 1983; Hawkins et al., 1983; Walters and Byrne, 1983; Walters, 1987a,b; Billy and Walters, 1989a; Lin et al., 2010). Other likely contributors to site-specific sensitization in *Aplysia* are NMDA-receptor-dependent long-term synaptic potentiation (LTP) of the activated synapses between nociceptors and motor neurons (Lin and Glanzman, 1994; Murphy and Glanzman, 1997; Antonov et al., 2003) and activity-dependent, proteolytic generation of several active PKC fragments (PKMs) in nociceptors and their postsynaptic targets (Sutton et al., 2004; Farah et al., 2017; Hu J. et al., 2017).

Nociceptive sensitization has also been investigated in the snail, *Helix lucorum.* An aversive chemical stimulus, quinine solution, applied to the head evokes head withdrawal and enhances subsequent withdrawal responses to mechanical and chemical test stimuli for several days, paralleled by potentiation of synapses onto withdrawal command neurons from mechanosensory and chemosensory neurons, along with hyperexcitability of the command neurons (Nikitin and Kozyrev, 1996). This sensitization involves many of the mechanisms described for general and site-specific sensitization in *Aplysia*. These include potentiating roles for 5-HT, cAMP, PKC, and C/EBP (Shevelkin et al., 1998; Nikitin and Kozyrev, 2000, 2005b; Nikitin et al., 2005; Tagirova et al., 2009) and NMDA receptor-dependent LTP of synapses from sensory neurons activated by the noxious stimulus (Nikitin et al., 2002; Nikitin and Kozyrev, 2003).

### Peripheral Sensitization in Gastropod Molluscs

In mammals, one component of site-specific sensitization contributing to primary hyperalgesia is localized hypersensitivity of the peripheral receptive fields of primary nociceptors (Gold and Gebhart, 2010; Smith et al., 2013). In *Aplysia*, short-term sensitization of nociceptor fields occurs after peripheral injection of 5-HT into the same fields (Billy and Walters, 1989b). Sensitization was recognized by a reduction in the force threshold for eliciting a response during application of a series of increasingly stiff nylon (von Frey) filaments to the skin. Either tissue injury (deep incision through half of the tail) or strong shock applied to the tail produced a persistent decrease in threshold for mechanical activation of nociceptors with receptive fields bordering a site traumatized 1–3 weeks earlier, but no sensitization in nociceptors with distant receptive fields (Billy and Walters, 1989a). This study also found a long-term expansion of the receptive fields of nociceptors innervating the traumatized region, and evidence for collateral sprouting from neighboring fields. The receptive field alterations are likely to involve injury-induced growth of peripheral fibers, given that nociceptor axons are capable of impressive regenerative growth after injury produced by crushing the nerve innervating the tail of *Aplysia* (Steffensen et al., 1995). Importantly, prior to complete regeneration (before receptive fields are restored to their normal size), the regenerating nociceptors exhibit peripheral sensitization, which was manifested as reduced threshold for activation by von Frey filaments, and hyperexcitability expressed as afterdischarge of action potentials in response to these brief mechanical stimuli (Dulin et al., 1995). In addition, nociceptor sprouting was observed within central ganglia (Steffensen et al., 1995), perhaps contributing to the enhancement of synaptic transmission observed after peripheral neural injury (see below). Peripheral regenerative growth and collateral sprouting can increase the density of nociceptive terminals near the injury and thereby increase nociceptive sensitivity, which also should be increased by hyperexcitability occurring in individual peripheral processes. These complementary alterations may function to compensate for loss of peripheral innervation caused by traumatic injury (Billy and Walters, 1989a; Dulin et al., 1995), and to protect the animal by increasing somatosensory vigilance (especially to mechanical stimulation in the injured region) after the animal is made more vulnerable by the injury (Walters, 1991, 1994).

### Axotomy-Induced Alterations of the Nociceptor Soma in *Aplysia* Resembling Alterations Linked to Neuropathic Pain in Mammals

Deep tissue injury is likely to sever nociceptor axons. In mammals, peripheral axotomy of a sufficient number of somatosensory neurons leads to neuropathic pain, which has been associated with hyperexcitability of primary afferent neurons at both the site of axonal injury (the neuroma) and the distant soma (Baron, 2009; Devor, 2009; Gold and Gebhart, 2010; Walters, 2012; Ellis and Bennett, 2013; Smith et al., 2013). The tail incision that was first used to study peripheral sensitization in *Aplysia* nociceptors cut through the entire depth of the mid-tail region to the midline, transecting ∼100% of the axons innervating a distal quarter of the tail (Billy and Walters, 1989a). A less severe incision transecting ∼50% of the axons innervating this tail quadrant was used to investigate effects of deep tissue injury on the excitability of the nociceptor soma (located in a central ganglion ∼10 cm away). One to 2 weeks after partial tail cut, somata of nociceptors likely to have been axotomized exhibited LTH compared to nociceptors with uninjured receptive fields, which showed little difference from nociceptors tested from uninjured animals (Gasull et al., 2005). LTH was similar when Ca^2+^-dependent exocytosis of neuromodulators was blocked during testing, suggesting that *maintenance* of the LTH was independent of ongoing modulation by extrinsic neuromodulators and instead represented long-lasting intrinsic alterations. On the other hand, *induction* of somal LTH by the injury could have been caused by activity-dependent extrinsic modulation (because no local anesthetic was present during the incision to reduce neuromodulator release), which as discussed above is known to induce LTH of the nociceptor soma and hypersensitivity of peripheral terminals after tail shock (Scholz and Byrne, 1987; Walters, 1987a; Billy and Walters, 1989b).

Evidence that somal LTH also can be induced directly by injury to nociceptor axons came from studies in anesthetized *Aplysia* utilizing an *in vivo* nerve crush injury. Crushing the nerve with forceps transected all axons in the peripheral nerve that innervates the tail without severing the nerve sheath (Walters et al., 1991; Steffensen et al., 1995). This injury produced, after a delay of 1–2 days, LTH of the nociceptor soma and an increase in amplitude of EPSPs from axotomized nociceptors onto tail motor neurons (Walters et al., 1991). The delay was caused by retrograde axonal transport of molecular signals from the injury site to the ganglion (Gunstream et al., 1995), a conclusion supported by showing that injection of axoplasm collected from crushed nerves into the somata of nociceptors from uninjured animals also produced somal LTH (Ambron et al., 1995). Furthermore, somal LTH could be induced by transecting the neurites of isolated nociceptors growing in culture, showing that extrinsic signals such as 5-HT released at the time of nociceptor injury are not required to induce LTH (Ambron et al., 1996; Bedi et al., 1998). At least two of the axonally transported induction signals are protein kinases; one an unidentified kinase that phosphorylates the transcription factor, Elk1 (Lin et al., 2003), and the other the cGMP-activated kinase, PKG (Sung et al., 2004). While injection into the soma of high concentrations of cAMP (a major downstream signal of 5-HT in *Aplysia* nociceptors) can induce somal LTH (Scholz and Byrne, 1988), somal injection of cGMP was much more potent than cAMP. NO-cGMP-PKG signaling was found to be required for *induction* of LTH by a damaging stimulation sequence applied to the body wall (Lewin and Walters, 1999). On the other hand, continuing activity of PKA in the nociceptor soma was required for the *maintenance* of somal hyperexcitability after nerve crush (Liao et al., 1999b). Crush-induced somal LTH lasted as long as 41 days, but decreased significantly in animals showing recovery of a tail-evoked, centrally mediated siphon response when nociceptors regenerated into the tail, with some recovery of the reflex and normal excitability evident within 2 weeks of the nerve crush (Gasull et al., 2005). LTH of the nociceptor soma after nerve injury (Ungless et al., 2002), like somal hyperexcitability and perhaps action potential broadening in the presynaptic terminal observed acutely or persistently by 5-HT or cAMP (Klein et al., 1982, 1986; Scholz and Byrne, 1988; Goldsmith and Abrams, 1992), involves the closing of “S-type” K^+^ channels that are open at resting potential and may be members of the 2-pore domain K^+^ (leak) channel family (Patel et al., 1998).

### Axotomy-Induced Alterations in Axons of *Aplysia* Nociceptors Similar to Persistent Somal Alterations, and Their Surprising Ca^2+^-Independent Induction

Long-term hyperexcitability lasting at least 1 day is also exhibited by *Aplysia* nociceptor *axons* following nerve crush in an excised ganglion-nerve preparation (Weragoda et al., 2004). This hyperexcitability, manifested as a decrease in both axonal action potential threshold and accommodation, is highly localized, extending <2 mm along the proximal side of the crush site. Axonal LTH was not reduced when the nerve was crushed in saline containing 1% of the normal extracellular [Ca^2+^], which blocked detectable effects of any released neuromodulators, suggesting that axonal LTH is a direct effect of axotomy. Because transection can depolarize *Aplysia* axons for several minutes (Berdan et al., 1993; Spira et al., 1993), an interesting question was whether similar depolarization (to ∼0 mV, produced by 2-min exposure of a 1.5 cm nerve segment to elevated [K^+^]) might by itself induce axonal LTH. Depolarization-induced axonal LTH was produced in 1% [Ca^2+^] saline and, like the induction of long-term synaptic facilitation by 5-HT (Montarolo et al., 1986; Casadio et al., 1999), induction of axonal LTH by either depolarization or nerve crush required rapamycin-sensitive (TOR-dependent) protein synthesis in the same nerve segment (Weragoda et al., 2004). Unexpectedly, given the general importance of Ca^2+^ as a cellular transducer of depolarization, the depolarization-induced LTH, as well as short-term (minutes) and intermediate-term (hours) axonal hyperexcitability induced by 2 min of strong depolarization occurred equally well when all detectable Ca^2+^ signaling was prevented by chelation of virtually all extracellular and intracellular Ca^2+^ (Kunjilwar et al., 2009). These results suggest that axotomy directly induces localized axonal LTH by mechanisms involving local rapamycin-sensitive protein synthesis (see Price and Inyang, 2015, for discussion of similar signaling in mammalian nociceptors) and, at least in part, a surprising depolarization-activated pathway that does not require Ca^2+^ signaling. The same Ca^2+^-independent depolarization procedure applied to the ganglion containing nociceptor-motor neuron synapses potentiated EPSPs from 15 min to >24 h, indicating that this unconventional depolarization-activated pathway can induce synaptic LTP as well as axonal LTH (Reyes and Walters, 2010).

Axonal LTH, unlike somal LTH (Liao et al., 1999a) can also be induced by prolonged or repeated exposure of a nerve segment to 5-HT (which modulates but does not activate nociceptor axons) in the absence of injury to the segment (Weragoda and Walters, 2007). Axonal LTH was induced by 5-HT in 1% or 0.02% [Ca^2+^] saline, suggesting a direct, Ca^2+^-independent effect on the axons. This neuromodulator-induced LTH, like injury-induced and depolarization-induced axonal LTH, requires local rapamycin-sensitive protein synthesis. It thus seems likely that natural injuries severe enough to transect nociceptor axons produce LTH in injured and nearby uninjured nociceptor axons by multiple mechanisms, including depolarization-induced and possibly 5-HT-induced signaling within the axons. Sources of peripheral 5-HT after injury could be central neuroendocrine release into the hemolymph (Cooper et al., 1989; Levenson et al., 1999), release by peripheral axons from central serotonergic axons at the site of injury (Marinesco et al., 2004a; Jhala et al., 2011), and/or local release from hemocytes mediating inflammatory-like responses at the injury site (Clatworthy et al., 1994; Farr et al., 1999). As in mammalian neuropathic pain models (Ellis and Bennett, 2013; Walters, 2014), not only injury signals intrinsic to damaged axons but also multiple extrinsic (inflammatory and damage-related) signals may contribute to persistent sensitizing effects of peripheral nerve injury in gastropod molluscs.

### Inhibition of Nociceptive Responses in Gastropod Molluscs

Nociception elicits and sensitizes some defensive responses in *Aplysia* but at the same time inhibits competing behavioral responses, including defensive responses incompatible with those directly elicited by the noxious stimulus (Walters et al., 1981; Walters and Erickson, 1986; Illich et al., 1994; Acheampong et al., 2012). The strongest evidence for an endogenous chemical inhibitor of nociceptive behavior and nociception in *Aplysia* has been found for FMRFamide, a short neuropeptide that is found in several phyla. In *Aplysia*, FMRFamide suppresses responses of primary nociceptors and their downstream targets centrally (Belardetti et al., 1987; Mackey et al., 1987; Montarolo et al., 1988; Schacher and Montarolo, 1991) and peripherally (Billy and Walters, 1989b; Cooper et al., 1989), at least in part by decreasing the excitability and synaptic strength of the nociceptors.

Opioid systems have been claimed to exist in gastropod molluscs on the basis of numerous behavioral-pharmacological and immunohistochemical studies, as well as on some biochemical and molecular evidence (e.g., Kavaliers et al., 1983; Leung et al., 1986; Kavaliers, 1987; Carpenter et al., 1995; Cadet and Stefano, 1999; Achaval et al., 2005; Nikitin and Kozyrev, 2005a; Miller-Pérez et al., 2008). In *Aplysia*, application of met-enkephalin at low doses suppressed the gill-withdrawal reflex (Lukowiak et al., 1982; Cooper et al., 1989). However, the existence in invertebrates of opioid systems that are homologous and functionally similar to opioid systems in vertebrates is controversial (Dores et al., 2002; Dreborg et al., 2008; Mills et al., 2016). Opioids and FMRFamide-related neuropeptides have been suggested to originate from a common ancestral gene (Taussig and Scheller, 1986). Alternatively, the weak molecular similarities between opioids and FMRFamide-related neuropeptides (and other families) might reflect convergent evolution because of fundamental constraints on binding between peptides and certain types of receptor proteins rather than homology across neuropeptide families (Greenberg et al., 1988).

## Immediate Responses to Noxious Stimulation in Cephalopod Molluscs

Cephalopods (squid, cuttlefish, octopuses, and nautiloids) comprise far fewer extant species than do gastropods or bivalves. However, they boast the largest living invertebrate (the colossal squid, weighing half a ton) as well as the largest nervous systems of any animal except some species of mammals and birds. While today’s cephalopods are far less common than fish, which currently represent the majority of large marine predators, during the Paleozoic and Mesozoic eras cephalopods were dominant marine predators (Kröger et al., 2011). For neuroscientists, the squid giant axon is famous because it enabled the discovery of the basic mechanisms of the action potential (e.g., Hodgkin and Huxley, 1952), and fundamental discoveries were also made about mechanisms of neurotransmitter release at the squid giant synapse (e.g., Katz and Miledi, 1967). However, few scientists working in pain-related fields have investigated squid.

Defensive functions, usually related to visual stimuli, have been investigated extensively in cephalopods, especially camouflage (Langridge et al., 2007; Allen et al., 2010; Staudinger et al., 2013; Bedore et al., 2015; Panetta et al., 2017), escape jetting (Otis and Gilly, 1990; Preuss and Gilly, 2000; Huffard, 2006), and chemical defense (Derby et al., 2007, 2013). In contrast, little attention has been paid to cephalopod responses to noxious stimulation or injury of the body, although the squid giant axon has been used to study cellular reactions to injury (Fishman et al., 1990; Godell et al., 1997). Behavioral responses to noxious stimulation were first described systematically for the squid *Doryteuthis pealeii*. Minor injury produced by amputation of the distal third of one of the 10 arms of an unanesthetized squid immediately evoked escape jetting and ink release, followed by display of cryptic body patterns and settling of the body onto the substrate (Crook et al., 2011). Grooming of the injured arm (which occurs after similar injuries in mammals) was never observed. Recordings of afferent electrical activity from the fin nerve in an excised fin preparation during mechanical stimulation revealed a population of nociceptive fibers that fire preferentially in response to high intensity mechanical stimuli (Crook et al., 2013). Because neuronal somata are not present in the fin, these mechanosensory neurons appear to be primary nociceptors, with somata located somewhere within the CNS. Nociceptive behavioral and neuronal responses were also described in a small octopus, *Abdopus aculeatus*, which sometimes uses self-amputation (autotomy) of an arm as a defense. Crushing the middle of an arm with forceps usually induced immediate autotomy, and always evoked escape jetting and ink release (Alupay et al., 2014). Interestingly, unlike squid with injured arms, all the octopuses showed wound-grooming behavior, holding the injured arm in the animal’s beak for at least 10 min. Nociceptive afferent units were found in recordings from the proximal end of the axial nerve cord, but these might have been second- or third-order neurons from ganglia within more distal parts of the arms. Direct evidence for primary nociceptors was found in units recorded from the mantle nerve that were activated selectively by strong mechanical stimuli applied to the mantle (Alupay et al., 2014).

## Nociceptive Sensitization and Pain-Like States in Cephalopod Molluscs

Nociceptive sensitization was not described in any cephalopod until recently. Crook et al. (2011) showed that amputation of the distal third of one arm of an unanesthetized squid (*D. pealeii*) sensitized defensive responses (escape jetting, ink ejection) for at least 2 days after injury. Some site-specific cutaneous sensitization was evident near the injury site in blindfolded squid. However, equally strong general sensitization was revealed by similarly enhanced responses to the tactile test stimulus applied to a contralateral arm, the head, or mantle of both freely swimming and partially restrained squid. Squid are highly visual species, and the largest enhancement of defensive responses occurred in freely swimming squid without blindfolds *before* contact with the von Frey filament, showing that the arm injury produced long-lasting sensitization (hypervigilance) to visual stimuli (Crook et al., 2011). Injured squid also became more likely to join schools of other squid when exposed to predator cues (Oshima et al., 2016). The hypervigilance and increased tendency to “seek safety in numbers” are consistent with an injury-induced, anxiety-like state. A somewhat different pattern of behavioral sensitization was reported after arm injury in the octopus, *Abdopus aculeatus*. Crushing the middle of an arm produced site-specific sensitization to von Frey stimulation, but little general sensitization of defensive behavior was found and no hypervigilance to visual stimuli was reported (Alupay et al., 2014).

Peripheral injury in cephalopods can sensitize primary nociceptors to mechanical stimulation of their peripheral receptive fields. A crush injury to one fin produced both immediate and long-term (lasting at least 24 h) sensitization, observed as a decrease in mechanical threshold and an increase in the number of afferent action potentials evoked by stimulation with a moderate intensity von Frey stimulus (Crook et al., 2013). Like the behavioral sensitization found after arm injury (Crook et al., 2011), nociceptor sensitization was not specific to the injured region; similar sensitization was found in nociceptors innervating the contralateral fin, suggesting that widespread nociceptor sensitization contributes to general behavioral sensitization in squid. Long-term sensitization of nociceptor responses was also found after natural injuries produced by attacks from other squid. Unexpectedly, fin injury produced significant ongoing (apparently spontaneous) electrical activity in fin nociceptors both ipsilateral and contralateral to the injured fin (Crook et al., 2013). Spontaneous activity in probable nociceptors has not been reported previously in invertebrates, although it is not uncommon in persistent pain models in mammals (Djouhri et al., 2006; Devor, 2009; Walters, 2012; Odem et al., 2018). This persistent spontaneous activity in the periphery may drive continuing activity in the brain that produces hypervigilance. At the same time, spontaneous activity generated in widespread nociceptors can provide little or no information about the location of the injury. Knowing the injury location may be less important for squid than the basic information that they have sustained a significant injury and are in a dangerous environment (Crook et al., 2013). In the octopus, *Adopus*, arm injury also produced widespread activity in peripheral neurons, increasing evoked and spontaneous activity recorded from the axial nerve cord at the base of both the previously injured and uninjured arms excised from injured animals compared to those excised from uninjured animals (Alupay et al., 2014). Given the large number of neuronal cell types in the axial nervous system, this afferent activity could represent activity in interneurons and/or primary sensory neurons.

Experiments on squid nociceptors led to the first direct demonstration in any species of the adaptiveness of nociceptive sensitization. Local and general nociceptor sensitization were found to be prevented by locally blocking all neural activity during fin crush, which was accomplished by injecting isotonic MgCl_2_ into the site to be injured (Crook et al., 2013; see also Butler-Struben et al., 2018). This non-specific local block effectively anesthetized the squid at the injured site while also locally blocking motor function (for similar effects and mechanisms in *Aplysia*, see Walters, 1987a,b and discussion in Liao and Walters, 2002). The localized motor block was experimentally useful because local relaxation of the chromatophores indicated the very limited spread and rapid reversal of the effects of the injected MgCl_2_. To test the adaptiveness of nociceptive sensitization, isotonic MgCl_2_ was injected into an arm just before distal amputation, 6 h before exposing the squid to a natural predator – black sea bass – for 30 min while confined in a relatively large tank (Crook et al., 2014). Direct effects of the MgCl_2_ remained localized to the injected arm, dissipated long before introduction to the fish, and by itself failed to alter camouflage, escape jetting, or inking during the encounter, or the likelihood of pursuit, attack, and capture by the fish. Interestingly, although human observers could not discern any difference in the general appearance or behavior of injured and uninjured squid (regardless of whether the neural block had been given earlier), the injured squid were selectively targeted by the fish and captured more often than uninjured squid. The adaptiveness of nociceptive sensitization was revealed by greater mortality during the 30-min encounter in squid that had been anesthetized during injury (81%) compared to squid sensitized by injury without anesthesia (55%), or to uninjured squid given sham procedures with anesthesia (25%) or without anesthesia (20%). Thus, persistent nociceptive sensitization can be evolutionarily adaptive by enhancing survival of a previously injured animal during predatory attack.

## Immediate Responses to Noxious Stimulation in Crustaceans

The phylum Euarthropoda contains over 80% of living animal species, most of which are terrestrial insects, but also includes crustaceans, arachnids (spiders, ticks, and mites), and myriapods (millipedes, centipedes). Most marine arthropods are in the crustacean subphylum, which includes both the most massive arthropod (the American lobster, weighing over 40 pounds) and tiny copepods that may have the greatest biomass of any animal group on the planet. All arthropods have a hard, chitinous, often mineralized cuticle that provides protection and a firm exoskeleton for attachment of muscles. Defensive behaviors have been investigated extensively in arthropods. In crustaceans, these include neurobiological studies of escape behavior, especially in crayfish (e.g., Edwards et al., 1999), and ecological studies of inducible defenses, often using water fleas (Daphnia) (Tollrian and Leese, 2010; Herzog et al., 2016). Across all arthropods, far less research has been conducted on behavioral and neural responses to noxious somatosensory stimuli than on responses to visual, auditory, and chemosensory stimuli (e.g., Joseph and Carlson, 2015; Göpfert and Hennig, 2016; Knaden and Graham, 2016; Ter Hofstede and Ratcliffe, 2016; Tomsic, 2016).

Surprisingly, primary nociceptors have yet to be identified in any crustacean, although indirect evidence supports their existence. Earlier suggestions that crustaceans have sensory neurons that detect noxious stimuli came from the elicitation of vigorous defensive responses by electric shock applied to the hard exoskeleton of crayfish and crabs (Krasne and Glanzman, 1986; Lozada et al., 1988; Fossat et al., 2014). The aversiveness of the shock was suggested by learning experiments, in which crayfish or crabs would escape or avoid a chamber paired with shock (Denti et al., 1988; Kawai et al., 2004; Magee and Elwood, 2013), and by anxiety-like effects produced by shock (Fossat et al., 2014). Somewhat similarly, shock delivered within a shell of a hermit crab stimulated evacuation of the shell and promoted switching to a new shell (Appel and Elwood, 2009a,b). A possible caveat in some of these studies, however, was a lack of controls for the possibility that avoidance was produced by long-lasting repellent chemicals released from animals shocked in a conditioning chamber or shell. A more general caveat for all studies of aversive learning (including mammalian studies) is that aversion, although arguably the most important feature of human pain, is not equivalent to pain; electric shock might produce non-painful sensations an animal seeks to avoid, such as itch, or unpleasant but non-painful feelings such as the pins and needles sensation that low-intensity shock can evoke in humans.

Other noxious stimuli investigated in crustaceans include the injection of formalin (sometimes used to model inflammatory or arthritic pain in rodents) into the joint of the claw of a crab, which produced brief freezing, rubbing of the claw, autotomy of the claw, and guarding-like postures (Dyuizen et al., 2012). These responses lasted less than 10 min, and tests for persistent sensitizing effects of the formalin injection were not reported. A few studies have described the elicitation of defensive responses in crustaceans by more natural noxious stimuli, providing indirect evidence for functional nociceptors. These include grooming by prawns of antennae stimulated with low- or high-pH saline (Barr et al., 2008) (although another study failed to find such responses in three decapod crustaceans) (Puri and Faulkes, 2010); grooming-like responses, escape, and withdrawal after stimulation of the mouth or eyes of crabs with acetic acid (Elwood et al., 2017); and defensive responses to touching crayfish claws or antennae with a hot probe (Puri and Faulkes, 2015). Tentative electrophysiological evidence for crustacean nociceptors came from recordings of increased ongoing afferent activity in crayfish antennal nerves during application of hot saline (Puri and Faulkes, 2015). However, the observed increase in activity was modest and the small volume applied in the bath might not have been sufficient to heat antennal receptors to noxious levels. Thus, the observed neural responses might have been to warmth rather than intense heat that threatens imminent tissue damage. An interesting question is whether peripheral nociceptors homologous with, or functionally equivalent to, the class IV multidendritic nociceptors in insects (see below) are found underneath the exoskeleton of crustaceans.

## Nociceptive Sensitization and Pain-Like States in Crustaceans

Few crustacean studies have addressed sensitizing effects of noxious stimulation that persist for hours, days, or longer. An early study showed that amputation of both claws of crayfish sensitized tail-flip escape behavior elicited by tactile or visual stimuli for at least several days (Krasne and Wine, 1975). Long-lasting nociceptive sensitization produced by aversive shock has been implicated in hermit crabs which, 24 h after being shocked in their shell, approached and occupied new shells more rapidly than did previously unshocked crabs (Appel and Elwood, 2009a). Sensitization lasting hours was found for the crayfish lateral-giant-fiber-mediated tail flip response after repeated electric shock (Krasne and Glanzman, 1986), which was associated with long-term synaptic potentiation of chemical and electrical synapses onto the lateral giant command neuron (Edwards et al., 2002). Long-lasting sensitization of the crayfish escape system, like sensitization of defensive responses in *Aplysia* (see above), may involve actions of 5-HT (Schnorr et al., 2014). Another similarity to *Aplysia* (see Lewin and Walters, 1999; Sung et al., 2004) is potential involvement of NO, with increased activity of NO synthase being reported in the crab nervous system for at least 1 h after injection of formalin into the joint of a claw (Dyuizen et al., 2012). Extensive knowledge of the neural circuitry controlling escape behavior in crayfish (Edwards et al., 1999) should facilitate investigation of nociceptive alterations in crustaceans, but very little is known about mechanisms of short- or long-term nociceptive sensitization in any crustacean species. Similar to the earlier finding with squid (Crook et al., 2014), the evolutionary adaptiveness of nociceptive sensitization in a crustacean was indicated recently by demonstrating that noxious shock applied to small amphipods (*Gammarus fossarum*) increased anxiety-like sheltering behavior and reduced capture by predatory goldfish (Perrot-Minnot et al., 2017).

Evidence for opioid inhibition of nociceptive behavioral responses in crustaceans, based on injection of morphine, has been reported for a mantis shrimp (Maldonado and Miralto, 1982) and crab (Lozada et al., 1988; Maldonado et al., 1989; Valeggia et al., 1989). However, a later study found that the high concentrations of morphine used in these crustacean studies did not reduce pain-like responses to shock in crabs, and may instead have produced a transient general impairment of motor function (Barr and Elwood, 2011). Although opioid systems are reported in crustaceans (Leung et al., 1987; Martinez et al., 1988), controversy about the existence of opioid systems in any invertebrate taxa (Dores et al., 2002; Dreborg et al., 2008) suggests that more study is needed to establish whether opioid-mediated anti-nociceptive function occurs in this sub-phylum. On the other hand, potent neuromodulatory systems that strongly suppress nociceptive responses have been found in crustaceans (e.g., Krasne and Wine, 1975; Vu and Krasne, 1993), so it will be of interest to further define the neuromodulatory mechanisms involved and their relationships to those described for anti-nociceptive systems in other animal groups.

## Immediate Responses to Noxious Stimulation in Insects

Insects have been the subject of numerous neurobiological studies of escape behavior (Camhi and Levy, 1988; Hoy et al., 1989; Allen et al., 2006; Card, 2012; Yager, 2012) and of chemical defenses (Sobotník et al., 2010; Nouvian et al., 2016; Touchard et al., 2016). Until recently, little attention was paid to injury-related behavior or to primary nociceptors in insects. Because they are relatively small, often very short-lived, and are reported to continue normal activities such as feeding or mating without interruption even as they sustain mortal injury, it has often been assumed that nociceptive systems are rudimentary and that pain-like states are absent in insects (Eisemann et al., 1984).

As in molluscs and crustaceans, some of the early experimental evidence for nociceptive responses came from experiments on learning in which electric shock was observed to elicit immediate withdrawal and escape responses as well as aversive learning (Horridge, 1962; Pritchatt, 1968; Booker and Quinn, 1981; Eisenstein et al., 1985). An early report described defensive responses of several lepidopteran larvae (caterpillars) to sharp mechanical stimuli, which included withdrawal, striking at the stimulation site with the head and mandibles, and non-directed thrashing of the body (Frings, 1945). The same responses, as well as cocking before striking (a preparatory posture to increase the force of the strike), regurgitation, and grooming-like behavior directed at a wound were analyzed in detail in larvae of the large moth, *Manduca sexta*, in response to stimulation with stiff filaments and sharp pinch (Walters et al., 2001). Striking and prolonged thrashing were described in the field during natural attacks by an avian predator, and similar striking responses were noted in wild lepidopteran larvae during egg-deposition attempts by parasitoid wasps (Walters et al., 2001). Tiny *Drosophila* larvae also show a well-studied nocifensive response – vigorous corkscrew-like rolling elicited by sharp mechanical stimuli or noxious heat (Tracey et al., 2003). Like the strike response of lepidopteran larvae, the rolling response of fruit fly larvae is evoked by attacks from parasitoid wasps, especially when the cuticle is penetrated, and this response was demonstrated to be adaptive by promoting escape from attacking wasps (Hwang et al., 2007; Robertson et al., 2013).

Primary nociceptors have been identified in both *Manduca* and *Drosophila*. A subset of sensory neurons with peripheral cell bodies and profuse multidendritic arbors beneath the epidermis and cuticle was discovered and shown to respond preferentially to strong mechanical stimuli in *Manduca* larvae (Grueber et al., 2001). The vast set of experimental genetic tools available for research on *Drosophila* has encouraged intensive research on apparently homologous nociceptors in fruit fly larvae (Grueber et al., 2002). These multidendritic class IV nociceptors were shown to be required for rolling responses to heat, sharp mechanical stimuli, and attacks by parasitoid wasps, and also for aversion to dry substrates (Tracey et al., 2003; Hwang et al., 2007; Johnson and Carder, 2012). Extracellular recordings showed heat-evoked activity in nerves containing axons of the nociceptors (Tracey et al., 2003). Optogenetic activation of this class of multidendritic sensory neurons was sufficient to trigger rolling behavior, and genetically targeted RNA interference (RNAi) silenced the nociceptors and prevented rolling responses (Hwang et al., 2007). The nociceptors express an ion channel in the TRPA family, “Painless,” that is distantly related to TRPA1 in vertebrates, and this channel is necessary for the defensive responses evoked by heat, harsh mechanical stimuli, and wasp attacks, and for aversion to dry substrates (Tracey et al., 2003; Hwang et al., 2007; Johnson and Carder, 2012). Class IV nociceptors also express TRPA1 (a close homolog of mammalian TRPA1), which contributes to noxious heat detection (Neely et al., 2011; Zhong et al., 2012), and they express at least two widely conserved channel types associated with mechanical nociception: degenerin/epithelial sodium channels (Zhong et al., 2010; Gorczyca et al., 2014; Mauthner et al., 2014) and a mechanosensitive piezo channel (Kim et al., 2012). Targeted silencing, optogenetic activation, and electron microscopy have revealed some of the downstream neural circuitry of interneurons and motor neurons that mediate rolling and other defensive responses in *Drosophila* in response to activation of identified nociceptors (Hu C. et al., 2017; Yoshino et al., 2017; Burgos et al., 2018). A separate class (III) of multidendritic sensory neurons was found to mediate cold nociception, involving three different TRP channels (Turner et al., 2016).

A mammalian transcription factor, PRDM12, known to control the developmental specification of primary somatosensory neurons and linked to nociceptive function in humans (Nagy et al., 2015), has a homolog, Hamlet, in *Drosophila* that specifies the fly multidendritic sensory neurons embryonically (Moore et al., 2002). This intriguing finding indicates that the development of insect and human nociceptive sensory neurons involves a shared regulatory gene inherited from an extremely ancient metazoan ancestor. RNAi knockdown of Hamlet in *Drosophila* or knockdown of some of its target genes reduced the sensitivity of larvae to noxious heat and decreased dendritic branching of the Class IV nociceptors (Nagy et al., 2015). Remarkably, overexpression in *Drosophila* nociceptors of PRDM12 mutants associated with impaired pain function in humans also impaired the larval response to noxious heat (Nagy et al., 2015). Another example of a conserved protein that was found to have similar functions related to noxious heat sensitivity in *Drosophila* and mammals is an auxiliary subunit, α2δ3, of voltage-gated Ca^2+^ channels (Neely et al., 2010). These results point to the conservation of some very basic molecular mechanisms tied to nociceptive function over at least 550 million years of evolution.

## Nociceptive Sensitization and Pain-Like States in Insects

The first description of nociceptive sensitization produced by a stimulus other than artificial electric shock in any arthropod came from studies of *M. sexta* larvae (Walters et al., 2001). Incremental sensitization of directed strike responses occurred during repeated sharp pinch but not gentle pokes delivered to one or more prolegs. General sensitization was also seen, lasting for up to an hour and expressed by an increased number of strikes during a series of gentle pokes applied to prolegs ipsilateral or contralateral to prolegs previously stimulated by multiple pinches. General sensitization after just a single noxious pinch of a proleg was later shown to be expressed as a marked decrease in strike threshold to mechanical stimulation, which persisted for at least 19 h (McMackin et al., 2016). This robust sensitization survived dissection after *in vivo* pinch, which allowed neural correlates of the sensitization to be examined (Tabuena et al., 2017). In contrast to nociceptive sensitization in *Aplysia* and mammals, neural sensitization did not include enhancement of primary afferent activity evoked by test stimulation of the previously pinched region, but it was expressed as increased evoked activity recorded from an interganglionic connective. This shows a form of central sensitization, perhaps with some functional and mechanistic similarities to the central sensitization that contributes to pain states in mammals (Woolf, 2011). Clues about the mechanisms of pinch-induced central sensitization came from blocking the induction of sensitization of strike responses (and central hyperactivity) by pre-treatment with NMDA receptor blockers, and reversal of sensitized strike responses by post-treatment with a blocker of cAMP-activated HCN channels (Tabuena et al., 2017). This pattern is interesting because, like some forms of nociceptive sensitization in *Aplysia* and mammals, it suggests that NMDA receptor-dependent LTP is involved in the induction of sensitization, and ongoing generation of cAMP may be involved in the maintenance of sensitization (e.g., Bavencoffe et al., 2016).

In insects (especially *Drosophila*, but also honeybees), as with gastropods and crabs, early indirect evidence for long-lasting effects of noxious stimulation came from studies of aversive learning and memory (e.g., Booker and Quinn, 1981; Busto et al., 2010; Diegelmann et al., 2013; Tedjakumala and Giurfa, 2013), including “pain relief learning” in which flies learned that a stimulus predicts safety from shock (Gerber et al., 2014). Intriguingly, a form of aversive operant learning potentially similar to conditioned place aversion was found in honeybees, in which flight of harnessed bees toward a salient landmark was punished by focused heat (Heisenberg et al., 2001). Direct studies of nociceptive sensitization have already yielded rich molecular insights. Epidermal damage and apoptosis caused by UV radiation without apparent injury to underlying nociceptors were associated with a long-lasting (∼1 day) sensitization of heat-evoked rolling responses (Babcock et al., 2009). The sensitization was expressed as both an enhanced incidence of rolling to focal contact with a probe heated to a noxious temperature (“hyperalgesia”) and by a decrease in the threshold temperature to elicit rolling (“allodynia”). Genetically targeted RNAi manipulations indicated that the allodynia required activation of a caspase in the epidermis with consequent signaling via *Drosophila* homologs of TNFα in epidermal cells and TNF receptor in adjacent nociceptors. Epidermal apoptosis turned out not to be necessary for sensitization, but downstream targets of TNF binding in nociceptors were, including a pathway involving p38 MAPK, NFκB, and a nuclear transcriptional regulator, enhancer of zeste (Jo et al., 2017).

A surprising discovery was that both thermal allodynia and hyperalgesia in larvae required a developmental signaling protein, the morphogen Hedgehog (Hh), a finding that inspired experiments by these authors using rats that provided the first evidence that the vertebrate morphogen homolog, sonic hedgehog, contributes to inflammatory and neuropathic pain in mammals (Babcock et al., 2011). In *Drosophila*, the thermal allodynia was found to depend upon TRPA (Painless) function, whereas thermal hyperalgesia depended on TRPA1 function in the same nociceptors (Babcock et al., 2011). Heat allodynia was found to require signaling by a tachykinin neuropeptide which, unlike substance P in mammals, is not produced by primary nociceptors (Im et al., 2015). Instead, UV radiation appears to stimulate the release of tachykinin from central neurons, which then binds to G protein-coupled tachykinin receptors in nociceptors, where it stimulates release of Hh, which by autocrine actions increases the expression and/or function of TRPA (Painless) channels and thereby causes allodynia. A critical pathway downstream from Hh in nociceptors is the bone morphogenetic pathway (BMP), which is required for nociceptive sensitization but not for normal nociception or nociceptor development in *Drosophila* (Follansbee et al., 2017).

Little or no evidence is available for endogenous antinociceptive systems in insects. Genes that are clearly homologous to opioid or opioid receptor genes in humans were not found in the *Drosophila* genome (Kreienkamp et al., 2002). While FMRFamide, which is antinociceptive in *Aplysia*, also occurs in insects, no links of this neuropeptide to inhibition of nociceptive responses have yet been implicated in arthropods (e.g., Merte and Nichols, 2002).

## Implications for the Evolution of Functions and Mechanisms Important for Nociceptive Sensitization and Pain

Paraphrasing the widely accepted definition of pain stated in the Introduction, pain is an aversive emotional experience related to actual or imminent tissue damage. A premise of this article is that mechanisms important for pain evolved from mechanisms that (1) have functioned to detect and evaluate tissue damage (nociception) and (2) to motivate adaptive behavior that would help avoid or minimize probable tissue damage (nociceptive sensitization). Molluscs and arthropods have provided abundant information about general functions and mechanisms of nociception and especially of nociceptive sensitization. Before discussing the implications of these findings, it should be noted that these animal groups are not optimal for answering all basic questions about the biology of pain. For example, questions related to the emotional content of pain can be addressed more clearly with mammalian species in which the expression of pain-related emotions appears similar to human expression (Darwin, 1886; Williams, 2002; Damasio and Carvalho, 2013). As another example, more has been learned about fundamental molecular mechanisms of nociceptive sensory transduction from the extremely simple, genetically tractable nematode, *Caenorhabditis elegans*, than from any other species, even genetically tractable *Drosophila* (Tobin and Bargmann, 2004; Venkatachalam et al., 2014; Katta et al., 2015). However, the molluscan and arthropod species discussed here have many analytic advantages, and in terms of numbers of neurons and some prominent physiological properties (notably, a reliance on classical overshooting action potentials for neural communication) (Lockery and Goodman, 2009) their nervous systems appear more similar to mammals than to *C. elegans*.

### Evolutionary and Functional Considerations

The large taxonomic separation between chordate, molluscan, and arthropod phyla means that similar functions and mechanisms found across these taxa represent either convergent evolution (homoplasy) or highly conserved descent from the last common ancestor of these groups (homology). This common ancestor is now thought to have lived more than 550 million years ago, near the end of the Ediacaran Period, when the complexity of animals, their nervous systems, and behavior began to increase quickly in response to substantial changes in the marine environment and the appearance of predators (Telford et al., 2015; Kristan, 2016; Budd and Jensen, 2017). It seems likely that the evolution of nociception, nociceptive sensitization, and pain-like responses, like other defensive responses, has been shaped by strong selection pressures exerted by predation (Vermeij, 1987; Walters, 1994; Huntley and Kowalewski, 2007; Crook et al., 2014; Monk and Paulin, 2014; Budd, 2015; Kristan, 2016). Similarities in nociception- and pain-related processes across all three phyla may point to common, independently derived solutions to general problems related to avoiding and surviving traumatic injury in a hostile environment. If effective solutions (and/or molecular building blocks that later proved effective for these solutions) had already evolved in the last common ancestor of chordates, molluscs, and arthropods, then some of these early solutions might have been conserved to function in today’s species. A fascinating finding consistent with this possibility is the common involvement in *Drosophila* and mammals of the PRDM family of transcription factors in both the embryonic development of nociceptive sensory neurons and in nociceptive responsiveness or pain (Nagy et al., 2015).

Many similarities in immediate responses to noxious stimulation are obvious in molluscs, arthropods, and chordates. Damaging or potentially damaging stimulation of the body (or electric shock that is likely to activate the fibers of primary nociceptors) usually elicits rapid defensive responses in nearly all animals tested across the animal kingdom, including all the molluscs and arthropods discussed here. The types of defensive responses vary enormously, depending upon the size, mobility, structure (including armor), developmental stage (larval versus adult), habitat, and life style of the species.

The prevalence of defensive responses to intense stimulation of the body surface supports the universal and obvious presumption that acute activation of nociceptive systems is an adaptive response to somatosensory stimuli threatening the integrity of the body (Sherrington, 1906; Kavaliers, 1988; Walters, 1994; Tobin and Bargmann, 2004; Costigan et al., 2009; Smith and Lewin, 2009; Burrell, 2017; Tracey, 2017; Sneddon, 2018). However, evolutionary adaptiveness is defined by reproductive success, not by survival; avoiding mortal danger is only adaptive to the extent that it enhances successful reproduction (Stearns and Medzhitov, 2015). In certain physiological states or at some stages of life it can be adaptive to lack, suppress, or ignore nociceptive sensation, an idea familiar to pain researchers because of the powerful suppression of nociceptive responses and pain by opioid and non-opioid systems during human fight-or-flight situations (Wall, 2002). Indeed, possession of opioid systems that strongly inhibit nociceptive responses has often been considered evidence for the possible existence of pain-like states in non-human species (Bateson, 1991; Sneddon et al., 2014). While the activation of opioid systems plays a large role in suppression of pain-related responses in mammals, it remains unclear whether homologous opioid systems function similarly or even exist in molluscs and arthropods (Dores et al., 2002; Kreienkamp et al., 2002; Dreborg et al., 2008).

Trade-offs between survival and reproductive success are found in all animal groups but seem especially striking in insects. It is well known that nociceptive responses fail to deter male mantids from mating with females that practice sexual cannibalism (Schwartz et al., 2016), and there are innumerable observations of adult insects showing no obvious changes in behavior after severe injury, e.g., continuing to use badly damaged limbs, copulating or eating while being eaten, or even eating their own innards spilled by abdominal rupture (Eisemann et al., 1984; Adamo, 2016). In contrast to these observations on adults, examination of larvae of *Drosophila* and *Manduca* has revealed specialized nociceptors that cover the entire body wall, and these larvae show strong, relatively long-lasting (hours or days) nociceptive sensitization of defensive behaviors evoked by mechanical or heat stimulation.

It would not be surprising for adults of short-lived species like most insects to maximize reproductive activities at the expense of behavior (such as nociceptive sensitization) that promotes survival of the adult but diverts energy and time away from mating and reproduction (Stearns and Medzhitov, 2015). On the other hand, some arthropods, such as lobsters, can live for several decades or longer. Lobsters have not been tested explicitly for nociceptive sensitization, and it is possible that the strong armor of large adults might reduce the need for such sensitization. However, claw amputation sensitizes escape behavior in crayfish (Krasne and Wine, 1975) – a much smaller but closely related decapod crustacean – and adult lobsters are reported to change their preferred defensive response from retaliation to escape after loss of their claws (Lang et al., 1977). It will be interesting to test lobsters and other long-lived crustaceans for long-lasting nociceptive sensitization after actual injury or events threatening damage that would increase vulnerability to predators and decrease future reproductive success.

Long-lasting nociceptive sensitization (lasting hours, days, or weeks) is expressed readily in molluscs such as *Aplysia* and squid. With lifespans of 1–2 years, these animals are not long lived, but their adult lives are substantially longer than many insects, and they grow much larger than insects. The high susceptibility to traumatic injury of their soft bodies and their well-developed capacities for repair and regeneration after injury sustained as adults (Dulin et al., 1995; Moffett, 2000; Shaw et al., 2016; Imperadore et al., 2017) coupled with documented risks of injury from predators (and in the case of squid, from conspecifics) (Nolen et al., 1995; Kicklighter et al., 2005; Watkins et al., 2010; Crook et al., 2013; Hanlon and Messenger, 2018) seems likely to make long-lasting nociceptive sensitization evolutionarily adaptive in these species. This prediction was confirmed in experiments on squid that provided the first demonstration that a procedure preventing nociceptive sensitization during injury reduces survival during subsequent exposure to a natural predator (Crook et al., 2014). Similar evidence for the adaptiveness of nociceptive sensitization came from showing that noxious shock delivered to an amphipod crustacean increased anxiety-like sheltering behavior and thereby reduced capture by a fish predator (Perrot-Minnot et al., 2017).

### Comparative Mechanistic Considerations

At physiological and molecular levels, as well as behavioral levels, the similarities of nociceptive sensitization across molluscs, arthropods, and chordates is striking. Each group shows enhanced defensive responses after noxious stimulation, and the behavioral sensitization is often associated with enhanced function (hyperexcitability, hypersensitivity, synaptic potentiation, growth) in primary nociceptors. Possible exceptions are crustaceans, where nociceptors have not yet been identified, and perhaps *Manduca* larvae, where prominent sensitizing effects of pinch have been found in central neural activity but not primary afferent activity (Tabuena et al., 2017). The neurophysiological alterations in nociceptors and their synaptic targets that have been identified in gastropods and insects involve signaling pathways known to contribute to the induction and maintenance of persistent pain in mammals.

Interestingly, the pathways identified in *Aplysia* and *Helix* are largely different from those identified in *Drosophila*, even though both sets are important in mammalian pain models. In addition to differences in focus between fields with different experimental traditions, this difference in pathways may reflect differences in the noxious stimuli employed in each model: primarily shock, nerve injury, or 5-HT application in *Aplysia* versus UV irradiation in *Drosophila*. Major pathways identified in *Aplysia* (and to a large extent in *Helix*) include G protein-coupled receptor- and NMDA receptor-driven signaling involving cAMP, PKA, PKC, and other protein kinases, regulation of gene expression by transcription factors such as CREB and C/EBP, regulation of mRNA translation by kinases such as TOR, and epigenetic regulation by non-coding RNAs. Major pathways identified in *Drosophila* include TNFα-p38 MAPK-NFκB, enhancer of zeste, Hedgehog, and BMP. In contrast, nociceptive sensitization in another insect, *Manduca*, induced by a different noxious stimulus, sharp pinch, was blocked by inhibitors of cAMP-activated ion channels and NMDA receptors (resembling effects in *Aplysia*). This suggests that differences between gastropods and insects in the pathways found thus far to be involved in nociceptive sensitization reflect at least in part differences in the noxious stimulation models employed rather than fundamental differences between the phyla in the cellular signaling underlying nociception-related neuronal plasticity.

Many of the pathways discussed here and others (e.g., growth factor-regulated pathways) implicated in nociceptive sensitization across major phyla are also important both for pain and for learning and memory (Walters and Moroz, 2009; Rahn et al., 2013; Géranton and Tochiki, 2015; Price and Inyang, 2015), although some features of epigenetic mechanisms in *Drosophila* have been noted to differ from mammals (and implicitly from molluscs) (Deobagkar, 2018). Widely shared molecular contributors to neural plasticity might represent conservation of fundamental mechanisms that originally were selected for adaptive responses to bodily injury (including nociceptive sensitization) and were later co-opted for learning and memory (Walters, 1991; Walters and Moroz, 2009; Price and Dussor, 2014). Alternatively, the original mechanisms could have evolved for learning and memory, later being co-opted for adaptive responses to injury. A possibility not mutually exclusive with the preceding two is that a set of core signaling modules evolved earlier for other functions and were pre-adapted for later use in plasticity underlying both injury/pain-related functions and learning-related functions (an example of what some evolutionary biologists call exaptation).

### Motivational and Emotional Considerations

During evolution, physiological and molecular mechanisms driving nociceptive functions became linked not only to sensory and discriminative processes that elicit immediate defensive responses, but also to motivational and cognitive processes that enable an animal to avoid ongoing and future threats related to a noxious experience. This requires an ability to maintain functional “awareness” of injury-induced vulnerability until the vulnerability subsides (perhaps until adequate repair of damaged body parts has been achieved). The phylogenetically widespread occurrence of memory of injury that may drive defensive motivational states is indicated by the examples of nociceptive sensitization described above in several molluscs and arthropods. Important support for this idea comes from the studies described in squid and amphipods in which nociception-induced, hypervigilant (anxiety-like) states reduce mortality from predators (Crook et al., 2014; Perrot-Minnot et al., 2017).

Interesting examples of nociception-induced hypervigilance also come from *Aplysia*. The general sensitization to tactile stimuli produced in *Aplysia* by unpredictable noxious shocks typically used to induce long-term general sensitization can be considered a hypervigilant state resembling the anxiety states in humans that motivate avoidance of general threats (Walters, 1980; Kandel, 1983). Interestingly, when repeated shock to *Aplysia* was predicted by pairing it with a non-threatening chemical cue, a conditioned fear-like state was triggered by subsequent exposure to the previously paired cue (Walters et al., 1981). This state was expressed as enhanced defensive responses (head withdrawal, tail withdrawal, inking, escape locomotion) and a suppressed appetitive response (feeding) in the presence of the chemical cue. Unlike the anxiety-like motivational state (ongoing general sensitization), the conditioned fear-like motivational state produced by similar amounts of shock was only expressed in the presence of the conditioned chemical cue, indicating that the same motivational state in *Aplysia* either can be persistently active if predictive cues are unavailable during noxious experience, or it can be activated selectively and transiently by subsequent detection of predictors of the noxious event (Walters et al., 1979, 1981; Walters, 1980; Colwill et al., 1988a,b). This indicates that gastropods have a capacity for cognitive processing of predictive information available during noxious experience. A similar capacity is suggested by higher-order aspects of aversive conditioning in *Drosophila*, such as learning about signals predicting safety from shock (Gerber et al., 2014).

Important questions remain about how invertebrates employ information from nociception and from nociception-associated stimuli to avoid further noxious stimulation. The ability to make adaptive choices on the basis of cognitive and motivational processing of information from noxious experience appears likely in many invertebrates, given that operant paradigms in which animals can freely choose among alternative responses reveal that noxious shock can produce diverse examples of avoidance. These include conditioned food aversion in gastropods (Mpitsos and Davis, 1973; Maximova and Balaban, 1984), conditioned avoidance of odors in *Drosophila* (Quinn et al., 1974), and avoidance learning in crabs (Fernandez-Duque et al., 1992; Magee and Elwood, 2013) and cockroaches (Pritchatt, 1968; Eisenstein et al., 1985) that may involve alterations in motor as well as sensory systems. Moreover, avoidance learning similar to conditioned place aversion produced by noxious heat in honeybees has been reported (Heisenberg et al., 2001). Conditioning of aversion to a place in which nociception had occurred previously and of preference to a place in which pain relief was experienced may be the clearest methods available for demonstrating the aversiveness of states hypothesized to be painful in animals (Minami, 2009; Navratilova et al., 2013). Increasingly used in mammalian pain studies (although the tests are still uncommon), conditioned place aversion and conditioned place preference tests could also help fill a large gap in the evidence implicating pain-like features of nociceptive sensitization states in invertebrates.

It seems likely that behavioral consequences of pain-like motivational states were major selection pressures for the evolution of mechanisms important for human pain. At some point(s) in evolution, physiological and molecular mechanisms driving the motivational and cognitive responses to actual or likely tissue damage became linked in some species to conscious emotional experience of pain. Building on the results reviewed here, it is likely that much more will be learned about mechanisms that not only detect and remember noxious experience, but also motivate a mollusc or arthropod to avoid further injury after significant nociception. Sensory and motivational mechanisms involved in nociceptive sensitization may also help to drive potentially separate processes that generate pain-like emotions in those animals that are capable of emotion. Whether any molluscs or arthropods have evolved a capacity for conscious emotion and for suffering after noxious experience remain major questions (see also Walters, 2018). While these questions are probably impossible to answer conclusively in species that are distantly related to humans (Allen, 2004), additional study of the nociceptive biology of molluscs and arthropods should point to commonalities and differences across these major phyla in selected biological characteristics that are important for producing pain and suffering in people.

## Author Contributions

The author confirms being the sole contributor of this work and approved it for publication.

## Conflict of Interest Statement

The author declares that the research was conducted in the absence of any commercial or financial relationships that could be construed as a potential conflict of interest.

## Footnotes

1 http://www.iasp-pain.org/terminology?navItemNumber=576#Pain
